# The Weight of Emotions in Decision-Making: How Fearful and Happy Facial Stimuli Modulate Action Readiness of Goal-Directed Actions

**DOI:** 10.3389/fpsyg.2018.01334

**Published:** 2018-08-02

**Authors:** Giovanni Mirabella

**Affiliations:** ^1^Istituto Neurologico Mediterraneo (IRCCS), Pozzilli, Italy; ^2^Department of Anatomy, Histology, Forensic Medicine and Orthopaedics, Sapienza University, Rome, Italy

**Keywords:** motor control, emotion, decision making, reaching arm movements, go/no-go task

## Abstract

Modern theories of behavioral control converge with the idea that goal-directed/voluntary behaviors are intimately tied to the evaluation of resources. Of key relevance in the decision-making processes that underlie action selection are those stimuli that bear emotional content. However, even though it is acknowledged that emotional information affects behavioral control, the exact way in which emotions impact on action planning is largely unknown. To clarify this issue, I gave an emotional version of a go/no-go task to healthy participants, in which they had to perform the same arm reaching movement when pictures of fearful or happy faces were presented, and to withhold it when pictures of faces with neutral expressions were presented. This task allows for the investigation of the effects of emotional stimuli when they are task-relevant without conflating movement planning with target detection and task switching. It was found that both the reaction times (RTs) and the percentages of errors increased when the go-signal was the image of a fearful looking face, as opposed to when the go-signal was a happy looking face. Importantly, to control for the role of the features of the stimuli, I ran a control task in which the same pictures were shown; however, participants had to move/withhold the commanded movement according to gender, disregarding the emotional valence. In this context, the differences between RTs and error percentages between the fearful and happy faces disappeared. On the one hand, these results suggest that fearful facial stimuli are likely to capture and hold attention more strongly than faces that express happiness, which could serve to increase vigilance for detecting a potential threat in an observer’s environment. On the other hand, they also suggest that the influence of fearful facial stimuli is not automatic, but it depends on the task requirements.

## Introduction

Decision making refers to the process of selecting an option from among a set of alternatives according to its probability of leading to best outcomes in terms of biological fitness. Critical to this executive function is the ability to accurately predict future outcomes (Mirabella, 2014; Mirabella and Lebedev, 2017). Nevertheless, living in a world where events cannot be predicted with certainty, agents must select actions based on limited information, i.e., they often must make risky decisions. Emotional information has a special weight in decision-making, as it automatically triggers adaptive behavioral modules selected during the course of evolution, driving agents to move toward appetitive goals while avoiding threats (Frijda, 1986; Frijda et al., 1989; Lang, 1995; Lang et al., 1997). Indeed, the ability to deal with emotional information is critical, because on the one hand it could prevent potential physical harm or unpleasant social interactions on the other hand it could promote physical pleasure or pleasant social interactions.

Although current results support the notion that emotional and motor processes are strongly interrelated, the empirical findings are often contradictory. Morrison et al. (2007) showed that in an emotional version of the go/no-go task, key releases were sped up and key presses were slowed down after subjects saw a video of a needle pricking a fingertip. In other words, pain observation modulated the motor system by speeding up withdrawal movements and slowing down approach movements of the finger. Other authors using emotional versions of go/no-go tasks found very different results (Berkman et al., 2009; Albert et al., 2010, 2012; Hartikainen et al., 2012; Zhang and Lu, 2012). Zhang and Lu (2012) found a decrease of reaction times (RTs) and greater accuracy during go-trials for both positive and negative images of faces with respect to neutral face images. Berkman et al. (2009) did not find differences in RTs or in accuracy between positive and negative images of faces. Albert et al. (2010), using some images from the International Affective Picture System (IAPS), did not find differences in the error rates both in go- and no-go trials, but they found that RTs were shorter for positive than for negative and neutral images. Interestingly, the same group (Albert et al., 2012) in a subsequent study with a similar design found that positive images elicited a larger number of errors than negative and neutral images, but RTs did not differ. Finally, Hartikainen et al. (2012), comparing the effect of drawing images of spiders versus neutral drawing images of flowers, found only an increase in error rates for negative stimuli during no-go trials.

Another group of studies followed another approach to assess whether emotional stimuli modulate action readiness, i.e., they evaluated the corticospinal motor tract excitability elicited by transcranial magnetic stimulation (TMS) delivered to the motor cortex (Avenanti et al., 2005; Coombes et al., 2005; Hajcak et al., 2007; Schutter et al., 2008; van Loon et al., 2010). Even this research provided contradictory results. On the one hand some studies showed that the presentation of unpleasant IAPS pictures (Coombes et al., 2005; van Loon et al., 2010) or of fearful facial expressions (Schutter et al., 2008) selectively increased corticospinal motor tract excitability and, as a consequence, the magnitude of the motor evoked potential (MEP) with respect to happy and neutral faces. However, on the other hand, Hajcak et al. (2007) found that the magnitude of the MEP was larger while participants viewed both pleasant and unpleasant images compared to neutral ones. In their view, this finding indicates that motor cortex excitability was increased by the arousal of the images instead of their valence (see also Baumgartner et al., 2007). Finally, Avenanti et al. (2005) observed decreased corticospinal motor tract excitability while participants were observing painful scenes with respect to non-painful scenes. An increase of motor cortex excitability is compatible with a decrease of RTs and vice versa, therefore the overall summary obtained from TMS studies closely resemble the contrasting results obtained using the emotional versions of the go/no-go task.

Clearly, differences in experimental designs can partially explain these contradictory results; for instance, it is likely that responses to painful stimuli might be different with respect to responses to fearful stimuli. In this vein de Valk et al. (2015) showed that anger fosters actions more efficiently than fear. However, in my view, the most important confounding factor of all these studies is that the emotional content of the stimuli was always incidental with respect to the task demands; i.e., emotional stimuli were irrelevant for task performance. Although it has been shown that even when emotions are task-irrelevant, they can influence motor behavior (Cacioppo and Gardner, 1999; Algom et al., 2004) it is very likely that their influences are highly subjective and variable. In two studies, Schulz et al. (2007, 2009) tried to overcome these limitations using the emotional valence of face images as an explicit cue for motor responses in a go/no-go task. In different blocks, participants were required to move on happy/sad faces and to withhold their actions on sad/happy faces. Unfortunately, the behavioral results of these two studies had discrepancies. Whereas in the first study, positive faces elicited faster responses with respect to sad faces (Schulz et al., 2007), in the second study this effect vanished (Schulz et al., 2009). A possible explanation lies in the fact that in the second study, neutral faces as well as emotional faces were employed. However, it has to be remarked that even this task design does not allow a direct comparison between the effects of negative and positive emotions on motor readiness, because in each experimental block, participants had to implement two different responses according to the stimulus valence, i.e., respond to one emotional stimulus category while withholding responses to the other. Therefore, in this context the emotional modulation of action readiness was conflated with task switching.

In order to directly compare equivalent decision-making processes underlying actions generation cued by emotional stimuli of different valence, I devised a new version of an emotional go/no-go task in which participants were required to move when emotional face stimuli with the same arousal values but with opposite valences were presented, and to withhold their responses on neutral faces. In addition, to control for the effects of stimulus features on motor readiness, I ran a control task where the same face stimuli were shown, but this time participants were requested to move according to the facial gender disregarding the emotional values.

## Materials and Methods

### Participants

Forty participants (20 males) took part in the study (mean ± SD age: 24.9 ± 2.9). All subjects were right-handed, as assessed with the Edinburgh handedness inventory (Oldfield, 1971), had normal or corrected-to-normal vision, and did not have a history of any neurological or psychiatric disorder. This study was approved by the institutional review board of Sapienza University of Rome and all experiments were performed in accordance with the ethical standards laid down in the Declaration of Helsinki of 1964. All participants gave their informed consent and none of them were informed about the purpose of the study.

### Stimuli

Stimuli consisted of grayscale pictures of faces of four adult actors (two males) taken from Pictures of Facial Affect (Ekman and Friesen, 1976). Each actor displayed three different facial expressions (fear, happiness, and neutral). Pictures were selected on the basis of their scores in arousal and valence as reported in the original database. In addition, at the end of the experimental session, each participant filled out a questionnaire in which the level of the arousal and of the emotional valence of each picture was evaluated. Arousal was evaluated on a 7-point scale (0 meant “no arousing” and 7 meant “high arousing”). The emotional valence was evaluated on a 15-points scale (-7 meant “very fearful”; 0 meant “neutral,” +7 “very happy”). The mean values and the corresponding standard deviations are reported in **Table 1**. Statistical analyses were performed to confirm, first, that positive and negative pictures were balanced with respect to their arousal levels, and second, that the pictures’ emotional valence was as assumed *a priori*. A two-way repeated measures ANOVA with arousal (levels: fear, happiness, and neutral) and sex (levels: male and females) as factors, revealed only a main effect of arousal [*F*(2,76) = 52.5, *p* < 0.0001]. *Post hoc* tests (pairwise comparisons with Bonferroni correction) showed that it was due to the fact that both fearful and happy faces have a greater arousal than neutral faces (all *p* < 0.0001) pictures, but the arousal of fearful and happy faces was not different (*p* = 0.56). The same ANOVA on emotional valence showed a main effect [*F*(1.58,60.2) = 1,047.1, *p* < 0.0001] and *post hoc* tests revealed that each of the three emotional categories were different from each other (all *p* < 0.0001).

**Table 1:** Mean value (±SD) of arousal and valence of three different facial expressions displayed in the pictures.

	Fearful faces	Happy faces	Neutral faces
Arousal	4.26 ± 1.3	4.49 ± 1.4	1.89 ± 1.6
Emotional valence	–4.66 ± 1.3	5.61 ± 0.9	–0.03 ± 0.5

All images were projected on a 17-inch PC monitor (CRT non-interlaced, refresh rate 75 Hz, 640 × 480 resolution, 32-bit color depth) on a black background at about 40 cm from the eyes of the participants. All images have the same dimension (5.8 cm × 7.4 cm or 8.25 × 10.9 degrees of visual angles, dva).

### Experimental Apparatus and Behavioral Tasks

In all experiments, participants were seated in a darkened and sound attenuated room, in front of a 17-inch PC monitor through which visual stimuli were presented. The PC monitor was coupled with a touch screen (MicroTouch; sampling rate 200 Hz) for touch-position monitoring. The temporal arrangements of stimulus presentation were synchronized with the monitor refresh rate. A non-commercial software package, CORTEX^1^, was used to control stimuli presentation and behavioral responses.

Participants were asked to perform two different versions of a go/no go task (the emotion and the gender discrimination task) in separate sessions. The order of administration of the two tasks was counterbalanced across subjects.

### Emotion Discrimination Task

**FIGURE 1:**
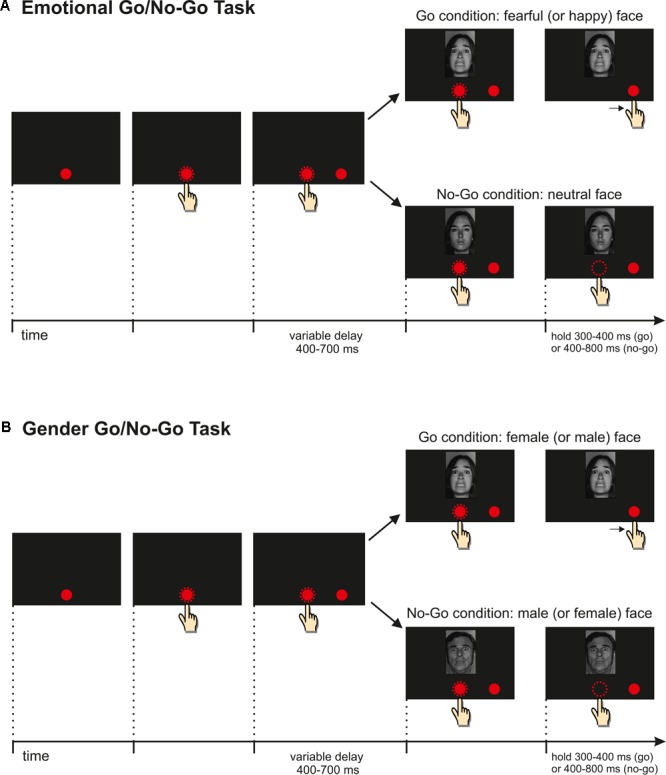
**(A)** Emotion-discrimination-task. The trial started with the presentation of a *red circle* at the center of the touchscreen. Subjects had to touch and hold it for a variable delay. Then a peripheral target appeared, followed by a picture depicting one of three expressions. Participants were instructed to reach and hold the peripheral target when the face expressed an emotion (fear or happiness) and to refrain from moving if the face had a neutral expression. All experimental conditions were randomized; **(B)** Gender-discrimination-task. The course of the events was the same described for the previous task; however, in this instance, participants were required to move when they saw a woman and not to move when they saw a man or vice versa. The order of administration of the two tasks was counterbalanced across subjects.

Each trial began with the presentation of a central red circle (2.43 cd/m^2^, diameter 2.8 cm or 4 dva) located 2 cm below the center of the screen, which participants were instructed to reach with their right index finger. As soon as the central target was touched, a peripheral red circle (diameter 2.8 cm or 4 dva) appeared to the right of the central target at an eccentricity of 8 cm or 11.3 dva. Subjects had to hold the central stimulus for a variable period (400–700 ms). Thereafter, the central stimulus disappeared and, concurrently, a picture of a face appeared just above the central stimulus (see **Figure 1A**). Whenever a face displayed an emotion, participants were required to reach as fast as possible the peripheral target and to hold it for a variable period of 300–400 ms. Conversely, when a neutral face was presented, participants had to withhold their response, holding the central stimulus for a variable period of 400–800 ms. Successful trials were signaled by acoustic feedback.

Each face was presented until 30 correct responses were given; thus the experiment consisted of 360 correct trials, and was run in three blocks (go trials frequency: 66%). Resting periods were allowed between blocks whenever requested. All experimental conditions were randomized. Error trials were repeated until participants completed the entire block. These trials were included in the statistic, but importantly they have not repeated right away, but randomly at a later point. This design was adopted in order to have an equal number of correct trials for each type of stimuli. To discourage participants from slowing down during the task, I set an upper reaction time limit for go-trials, i.e., every time RTs were longer than 500 ms, go-trials were signaled as errors and aborted (overtime reaching-trials, see Mirabella et al., 2006, 2008; Federico and Mirabella, 2014). Overtime reaching-trials were not repeated and were included in the analyses to avoid cutting the right tail of the RT distribution, and they accounted for 4.4% of the total go-trials.

### Gender Discrimination Task

The gender discrimination task has the same timing parameters and the same stimuli described for the emotion discrimination task, but it differs in the response demand (see **Figure 1B**). In fact, in the gender discrimination task, participants had to move according to the gender of the face. In order to avoid any gender bias, half of the participants had to move when faces of males were displayed and to withhold the movement when faces of females were shown, and the other half of participants had to perform this the other way around. This was because I wanted to keep the same frequency of go trials I had in the emotion discrimination task, i.e., 66%, the face stimuli indicating the go trials were presented until 40 correct responses were given, whereas face stimuli indicating the no-go trials were presented until 20 correct responses were given. As in the emotion discrimination task, error trials were repeated until performed correctly. Thus, this experiment also consisted of 360 correct trials, run in three blocks, and all experimental conditions were randomized. The upper reaction time limit for go-trials was set to 500 ms, overtime reaching-trials were not repeated and accounted for 3.3% of the total go-trials.

### Data Analyses

Reaction times of correct go trials and error rates were taken as behavioral parameters. RTs were determined as the time difference between the time of the occurrence of the go-signal, and the movement onset. Movement times (MTs) of correct go trials, computed as the time difference between time of movement onset and the time at which subjects touched the peripheral target, were also calculated. However, as the statistical analyses did not yield any effect, I do not report these results here.

Go-trials that had RTs longer than the mean plus three SDs, and those shorter than the mean minus three SDs were excluded from the analysis. In total 0.74% of the data was eliminated. Errors in go trials were defined as those instances in which participants kept their index finger on the central stimulus, instead of reaching the peripheral target. For each participant, the error rate was computed as the ratio between the number of errors in a given condition (e.g., fear in the emotion discrimination task), and the overall number of trials for the same condition (e.g., all go trials in which a fearful face was shown).

Two different three-way ANOVAs with a mixed design [between-subjects factor: Sex (male and female participant); within-subjects factors: Emotion (fear and happiness) and Task (emotion discrimination task and gender discrimination task)] were performed to analyze mean RT differences and mean error rates across experimental conditions. Bonferroni corrections were applied to all *post hoc* tests (pairwise comparisons).

In order to provide a measure of the effect-size, I computed partial eta-squared ($\eta_{p}^{2}$) for each ANOVA with values of 0.139, 0.058, and 0.01 indicating large, medium, and small effects, respectively, and Cohen’s *d* as the effect size for *t*-tests with values of 0.8, 0.5, and 0.2 indicating large, medium, and small effects (see Lakens, 2013). All data can be downloadable^2^.

## Results

**FIGURE 2:**
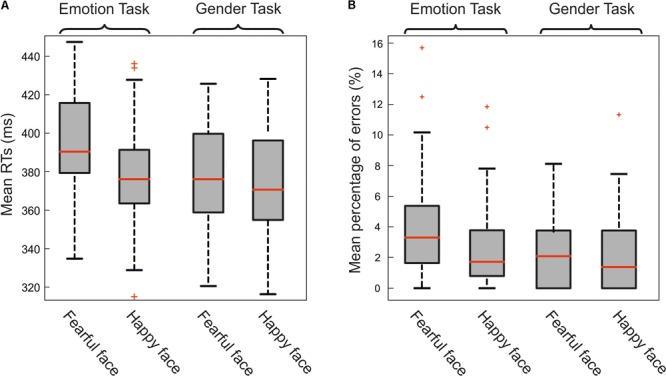
**(A)** Effect of emotional facial expression on reaction times (RTs). Mean RTs to fearful and happy emotional faces in the emotion-discrimination-task (on the *left*) and in gender-discrimination-task (on the *right*). Results were split according to the gender of the participants. Overall females were faster than males and, more importantly, participants were slower when the go-signal was a fearful face than when it was a happy face just during the emotion-discrimination-task (see text for the statistics). In each box plot, the boundary of the box closest to zero indicates the first quartile, a *red line* within the box marks the median, and the boundary of the box farthest from zero indicates the third quartile. Whiskers indicate values 1.5 times the interquartile range below the first quartile and above the third quartile. **(B)** Effect of emotional facial expression on the percentage of errors. Mean percentage of errors to fearful and happy faces in the emotion-discrimination-task (on the *left*) and in gender-discrimination-task (on the *right*). Participants made a larger amount of mistakes in the emotion-discrimination-task than in the gender-discrimination-task. No differences were found between males and females (see text for the statistics).

The three-way ANOVA on mean RTs of go-trials revealed several effects (**Figure 2A**). First of all, there was a main effect of Emotions (*F*[1,38] = 37.1, *p* < 0.0001, *M*_diff_ = 8.7 ms, 95% CI[5.8, 11.7]; $\eta_{p}^{2}$ = 0.49) due to the fact that participants moved faster after the presentation of a happy face (*M* = 376.8 ms; *SD* = 27 ms) than after the presentation of a fearful face (*M* = 385.5 ms; *SD* = 29.2 ms).

Second, there was a main effect of Task (*F*[1,38] = 11, *p* = 0.002, *M*_diff_ = 11.5 ms, 95% CI[4.5, 18.6], $\eta_{p}^{2}$ = 0.22) as participants moved faster during the gender discrimination task (*M* = 375.4 ms; *SD* = 27.7 ms) than during the emotion discrimination task (*M* = 386.9 ms; *SD* = 27.9 ms). These effects are qualified by the interactions between Emotions and Task (*F*[1,38] = 29.4, *p* < 0.0001, $\eta_{p}^{2}$ = 0.44). In fact, while during the emotional discrimination task the presentation of a fearful face significantly increased the RT lengths (*M* = 394.6 ms; *SD* = 28.2 ms) with respect to the presentation of a happy face (*M* = 379.3 ms; *SD* = 25.9 ms; *t*[39] = 8.5; *p* < 0.0001; *M*_diff_ = 15.3 ms, 95% CI [11.6, 18.9]; Cohen’s *d* = 1.2) during the gender discrimination task there was no difference between the RTs after a fearful face (*M* = 376.5 ms; *SD* = 27.3 ms) and after a happy face (*M* = 374.3 ms; *SD* = 28.1 ms; *t*[39] = 1.2; *p* = 0.24; *M*_diff_ = 2.5 ms, 95% CI [-1.6, 6.1]; Cohen’s *d* = 0.17). I also found that participants had slower RTs after the presentation of a fearful face in the emotional discrimination task than in the gender discrimination task (*t*[39] = 4.78; *p* < 0.0001; *M*_diff_ = 18 ms, 95% CI [10.4, 25.7], Cohen’s *d* = 1.4).

The same three-way ANOVA on the average rates of mistakes provided a similar pattern of results (**Figure 2B**). There was a main effect of Emotions (*F*[1,38] = 8.7; *p* = 0.005; *M*_diff_ = 0.83, 95% CI [0.26, 1.4]; $\eta_{p}^{2}$ = 0.19) given by the greater number of mistakes occurring after the presentation of a fearful face (*M* = 3.2; *SD* = 3.1) than after the presentation of a happy face (*M* = 2.4; *SD* = 2.8). There was also a significant interaction between Emotions and Tasks (*F*[1,38] = 5.8, *p* = 0.02, $\eta_{p}^{2}$ = 0.13). This was due to the fact that in the emotional discrimination task participants made more mistakes after the presentation of fearful faces (*M* = 4.1; *SD* = 3.6) than after the presentation of happy faces (*M* = 2.6; *SD* = 2.9; *t*[39] = 3.6; *p* = 0.001; *M*_diff_ = 1.5, 95% CI [0.6, 2.3]; Cohen’s *d* = 0.9). Conversely, during the gender discrimination task the error rate after the presentation of fearful faces (*M* = 2.4; *SD* = 2.3) was not significantly different from that recorded after the presentation of happy faces (*M* = 2.2; *SD* = 2.6; *t*[39] = 0.5; *p* = 0.64; *M*_diff_ = 0.17, 95% CI [-0.6, 0.9], Cohen’s *d* = 0.17). In addition, participants had a higher error rate after the presentation of a fearful face in the emotional discrimination task than in the gender discrimination task (*t*[39] = 2.8; *p* = 0.008; *M*_diff_ = 1.7, 95% CI [0.5, 3], Cohen’s *d* = 1.12).

## Discussion

The ability to generate appropriate responses (especially in social situations when a person has to make sense of the behavior of others and to establish appropriate social bonds with other people) relies heavily on the integration of emotional information with ongoing motor/cognitive processes. Impairments in this ability lead to psychiatric and neurological disorders characterized by deep alterations of interpersonal relationships (such as autism spectrum disorders, bipolar disorder anxiety, antisocial, and sociopathic personality disorders). Therefore, understanding how the interactions between emotion and cognition take place represents a milestone toward the comprehension of pathologies affecting social cognition. However, the relatively small bulk of studies tackling this topic provides contradictory or ambiguous results due to several confounding factors. The most important ones concern the aspects of the behavioral task employed (e.g., task requirements, stimulus modality, and lack of control experiments).

To overcome these limitations, I designed two tasks which provided measures of response readiness for exactly the same stimuli but under different rules, i.e., in the emotion-discrimination-task emotions are task-relevant whereas in the gender-discrimination-task emotions are task-irrelevant. The direct comparison between the results across the two tasks allowed to assess directly how the relevance of emotions affects action planning. Results were straightforward: only when the emotional content of the stimuli was relevant for the task it affected the generation of actions, i.e., fearful face expressions slowed down movement initiations and increased the number of errors with respect to happy face expressions. Conversely, when participants had to move according to the gender of the face the differences in RTs and error percentages between fearful ad happy faces disappeared. As I compared the responses elicited by the same stimuli across of the same participants in the two tasks, it can be concluded that these results cannot depend either on the variability of individuals or on the stimuli features. Instead, they suggest that fearful facial stimuli impact on arm reaching planning more than happy facial stimuli, provided that they are task-relevant. Finally, even though it is possible that participants visually inspected the same stimuli in different ways according to the task rules, i.e., they used different cues for detecting the emotion or the gender of the face stimuli, this still means that in the gender discrimination task the emotional valence of the stimuli is disregarded. Further studies using the eye-tracker are needed to clarify this issue.

### Fearful Task-Relevant Faces Increase Vigilance and Captures Attention

Faces convey information that is essential for appropriate social communication in humans (Blair, 2003). In particular, emotional facial expressions are crucial to infer the observed person’s feelings, state of mind, and intentions (Vuilleumier, 2005). Being salient stimuli, emotional facial expressions are capable of changing the course of actions biasing attention (Yiend, 2010). However, their effects are different according to the valence of the facial expression. Fearful faces signal a potential threat, and thus they tend to induce action preparations associated with fight–flight behavior (Anderson and Phelps, 2001; Schutter et al., 2008). Conversely, happy facial expressions tend to promote approach-related behaviors aimed to deal with rewarding stimuli (Blair, 2003). However, the net effect of emotional facial expressions on action preparation is controversial. For instance, it has been repeatedly shown that threatening expressions (e.g., fearful or angry faces), even when task-irrelevant, induce rapid action planning, which is thought to prepare the subject to face a potential threat (e.g., Schutter et al., 2008; van Loon et al., 2010). However, de Valk et al. (2015) in a task where participants were required to touch as quickly as possible pictures of angry, fearful, and neutral faces/bodies, found that while participants responded faster to angry than to neutral stimuli, no significant difference was observed between fearful and neutral stimuli. They interpreted their findings by suggesting that although fear and anger are both negative emotions, they indicate different sources of threat. Angry faces can be perceived by the observer as a direct threat toward him or her, requiring immediate action. In contrast, fearful faces could potentially indicate a potential threat in observer’s environment, leading to increased vigilance for detecting the source of danger (Whalen, 1998; Davis and Whalen, 2001), hence slowing down the speed of response and interfering with the ongoing action.

A consistent corpus of experiments indicates the existence of an attentional bias toward emotional stimuli (e.g., Lang, 1995; Yiend, 2010). Both positive (Pool et al., 2016) and negative (Bar-Haim et al., 2007) salient affective stimuli can capture attentional resources diverting processing away from the ongoing action, in order to respond to potentially advantageous or threatening stimuli. Even though some studies seem to indicate that the effect of positive and negative facial expression might be similar, at least under certain experimental contexts (Hodsoll et al., 2011; D’Hondt et al., 2013), there is a general agreement about the fact that attentional bias for positive stimuli is relatively modest even when compared with neutral stimuli (Pool et al., 2016). Differently, threatening information is thought to exert a more efficient capture of attentional resources (Fox et al., 2002; Vuilleumier and Huang, 2009) allowing a quick detection of potential threats and enabling appropriate responses (Öhman and Mineka, 2001). Such hypervigilance toward threatening cues makes it difficult to direct attention away from threat once it is detected (Weierich et al., 2008), and might also be exaggerated in patients suffering from phobia and anxiety (Ouimet et al., 2009). Nevertheless, on the basis of the results of de Valk et al. (2015), it could be argued that fearful stimuli could be particularly effective in capturing and holding attention interfering with ongoing actions more than other emotional stimuli. This account would explain why subjects were slower and made a greater amount of mistakes after the presentation of a fearful face than after the presentation of a happy face.

## Conclusion

The effects of fearful and happy faces on motor preparation were compared under two conditions, i.e., when they were task-relevant versus when they were task irrelevant. I found that just in the former case an interference effect occurred, as participants were slower and less accurate when the go-signal was a fearful face than when it was a happy face. These findings suggest that fearful facial stimuli capture and hold attention more strongly than faces expressing happiness, deeply interfering with reaching arm planning and execution. This might be a consequence of the fact that fearful facial stimuli are likely to signal environmental threats that are not directed to the observer but are present in his/her environment and thus they requires further exploration before planning actions. Crucially, when the emotional valence of the stimuli is not relevant for task performance, all effects vanished and no differences between fearful and happy faces occur. Even though further experiments are needed to replicate this finding (e.g., using different facial emotional expressions, such as anger or surprise), it indicates that, at least under my experimental conditions, the influence of fearful facial stimuli is not automatic. Here it is important to underline that my results are not in contrast with the findings showing that emotional stimuli, and in particular stimuli with negative valence, can be automatically processed by dedicated brain networks (Pegna et al., 2005; Van den Stock et al., 2014; Méndez-Bértolo et al., 2016; Burra et al., 2017; for a review see Diano et al., 2016). Differently, this research suggests that to obtain a reproducible behavioral effect, subjects must be aware of the emotional values of the stimuli.

## Author Contributions

GM conceived and designed the experiments; programmed the temporal arrangements of stimulus presentation using a non-commercial software package, CORTEX (https://www.nimh.nih.gov/labs-at-nimh/research-areas/clinics-and-labs/ln/shn/software-projects.shtml); collected the data, helped by a few undergraduate students who were performing their internship, they have all been acknowledged; analyzed the data, using self-made Matlab programs and IBM SPSS Statistics package; wrote the article; and made the figures.

## Conflict of Interest Statement

The author declares that the research was conducted in the absence of any commercial or financial relationships that could be construed as a potential conflict of interest. The reviewer MD and handling Editor declared their shared affiliation.

## Footnotes

1 https://www.nimh.nih.gov/labs-at-nimh/research-areas/clinics-and-labs/ln/shn/software-projects.shtml

2 https://osf.io/f3b54/
